# Crystal structure of 4-[(2*E*)-3-(4-meth­oxy­phen­yl)prop-2-eno­yl]phenyl benzoate

**DOI:** 10.1107/S1600536814018303

**Published:** 2014-08-16

**Authors:** S. Sathya, D. Reuben Jonathan, K. Prathebha, J. Jovita, G. Usha

**Affiliations:** a PG and Research Department of Physics, Queen Mary’s College, Chennai-4, Tamilnadu, India; b Department of Chemistry, Madras Christian College, Chennai-59, India

**Keywords:** crystal structure, benzoate, hydrogen bonding

## Abstract

In the title compound, C_23_H_18_O_4_, the meth­oxy­benzene ring and attached C=C grouping are disordered over two sets of sites in a 0.823 (5):0.177 (5) ratio. The dihedral angles between the central benzene ring and the pendant phenyl and meth­oxy­benzene ring (major orientation) are 51.21 (1) and 51.6 (1)°, respectively. In the crystal, inversion dimers linked by pairs of C—H⋯O hydrogen bonds generate *R*_2_^2^(28) loops.

## Related literature   

For background to flavenoids, see: Di Carlo *et al.* (1999 ▶); Rackova *et al.* (2005 ▶); Harborne & Williams (2000 ▶). For related structures, see: Moreno-Fuquen *et al.* (2014 ▶); Jasinski *et al.* (2011 ▶); Sathya *et al.* (2014 ▶).
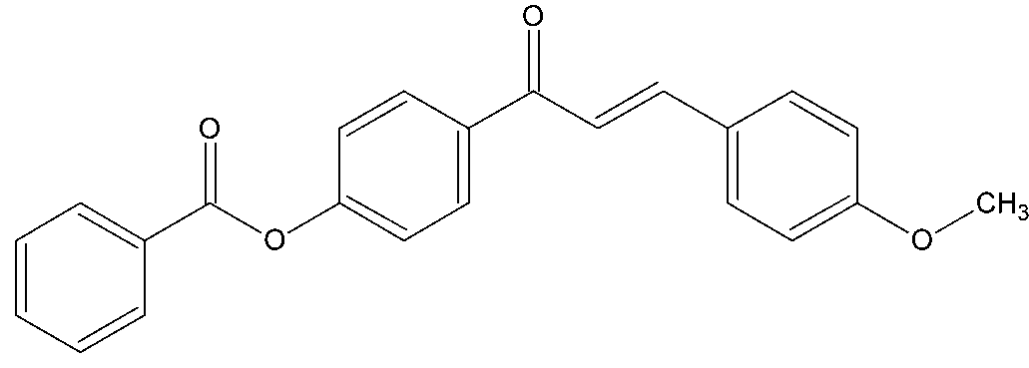


## Experimental   

### Crystal data  

C_23_H_18_O_4_
*M* *_r_* = 358.37
Monoclinic, 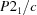

*a* = 20.146 (5) Å
*b* = 14.513 (5) Å
*c* = 6.187 (5) Å
β = 94.828 (5)°
*V* = 1802.5 (16) Å^3^
*Z* = 4
Mo *K*α radiation
μ = 0.09 mm^−1^
*T* = 293 K
0.35 × 0.30 × 0.25 mm

### Data collection  

Bruker APEXII CCD diffractometer
Absorption correction: multi-scan (*SADABS*; Bruker, 2004 ▶) *T*_min_ = 0.969, *T*_max_ = 0.978
17041 measured reflections
3170 independent reflections
2523 reflections with *I* > 2σ(*I*)
*R*_int_ = 0.045

### Refinement  

*R*[*F* ^2^ > 2σ(*F* ^2^)] = 0.056
*wR*(*F* ^2^) = 0.195
*S* = 1.11
3170 reflections
320 parameters
334 restraints
H-atom parameters constrained
Δρ_max_ = 0.21 e Å^−3^
Δρ_min_ = −0.17 e Å^−3^

### 

Data collection: *APEX2* (Bruker, 2004 ▶); cell refinement: *SAINT* (Bruker, 2004 ▶); data reduction: *SAINT*; program(s) used to solve structure: *SHELXS97* (Sheldrick, 2008 ▶); program(s) used to refine structure: *SHELXL97* (Sheldrick, 2008 ▶); molecular graphics: *ORTEP-3 for Windows* (Farrugia, 2012 ▶); software used to prepare material for publication: *SHELXL97*.

## Supplementary Material

Crystal structure: contains datablock(s) I, New_Global_Publ_Block. DOI: 10.1107/S1600536814018303/hb7255sup1.cif

Structure factors: contains datablock(s) I. DOI: 10.1107/S1600536814018303/hb7255Isup2.hkl

Supporting information file. DOI: 10.1107/S1600536814018303/hb7255Isup3.cml

**Fig. 1.**
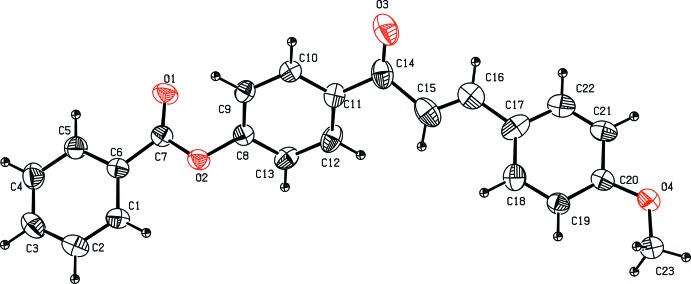
The molecular structure of the title compound, with displacement ellipsoids drawn at the 30% probability level.

. DOI: 10.1107/S1600536814018303/hb7255fig1.tif
The mol­ecular structure of the title compound, with displacement ellipsoids drawn at the 30% probability level.

**Fig. 2.**
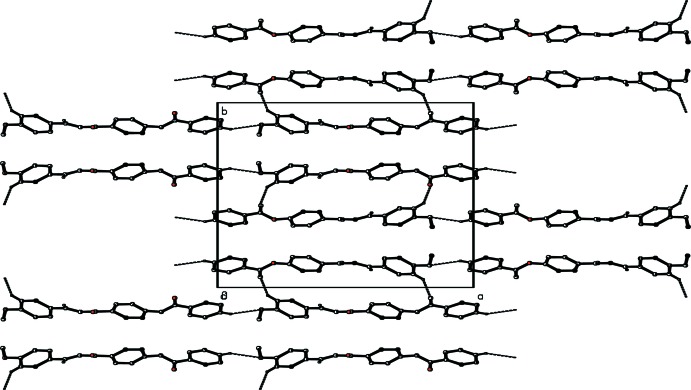
The packing of the molecules in the crystal structure. The dashed lines indicate the hydrogen bonds.

. DOI: 10.1107/S1600536814018303/hb7255fig2.tif
The packing of the mol­ecules in the crystal structure. The dashed lines indicate the hydrogen bonds.

**Fig. 3.**
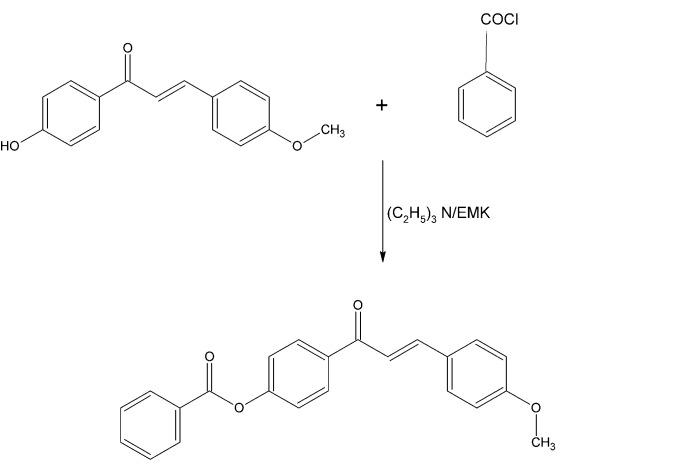
Experimental procedure.

. DOI: 10.1107/S1600536814018303/hb7255fig3.tif
Experimental procedure.

CCDC reference: 1018848

Additional supporting information:  crystallographic information; 3D view; checkCIF report

## Figures and Tables

**Table 1:** Hydrogen-bond geometry (Å, °)

*D*—H⋯*A*	*D*—H	H⋯*A*	*D*⋯*A*	*D*—H⋯*A*
C21—H21⋯O1^i^	0.93	2.56	3.280 (3)	135
C21′—H21′⋯O1^i^	0.93	2.65	3.546 (7)	163
C23′—H23*F*⋯O1^ii^	0.96	2.50	3.24 (7)	135

Symmetry codes: (i) 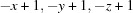; (ii) 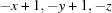.

## supplementary crystallographic information

### S1. Comment

Flavonoids belong to a large group of abundant plant secondary metabolites, which can be found in vascular plants such as ferns, conifers and flowering plants (Di Carlo *et al.*,1999). Natural and synthetic flavonoids are therefore of considerable interest in the development of new therapeutic agents for various diseases and are generally believed to be non-toxic compounds since they are widely distributed in the human diet (Rackova *et al.*., 2005; Harborne *et al.*, 2000).

In the compound, the phenyl benzoate and chalcone groups are linked by a phenyl ring (B). The C—O bond length 1.219 (6) Å indicate the double bond character. The C—O bond length of phenyl benzoate 1.403 (4) & 1.197 (4) Å, respectively, indicate the single and double bond characters and are comparable with similar reported structure [Moreno-Fuquen *et al.*,2014]. The bond angles C11—C14—C15(118.7 (5)°, C15—C16—C17(125.9 (5)°) are slightly shorter than the normal values but are comparable with those in similar reported structure [Sathya *et al.*,2014 & Jasinski *et al.*,2011]. This may be due to the presence of keto group and the associated steric forces. The prop-2-en-1-one group is twisted slightly with a C15—C16—C17—C22 & C12—C11—C14—O3 torsion angle of [167.8 (4)° & 155.5 (5)°], respectively, and are in good agreement with similar reported structures (Sathya *et al.*, 2014 & Jasinski *et al.*, 2011). The central ester moiety[O1/C7/O2] is twisted(C9—C8—O2—C7/C13—C8—O2—C7) away from the benzene ring on both sides by 59.5 (3)&-126.4 (3)°, respectively, and are comparable with similar reported structure (Moreno-Fuquen *et al.*,2014)

In the crystal, the symmetry related molecules linked by C—H···O type hydrogen bonds forming dimer *R*_2_^2^ (28), described by the graph set motif. These dimer units inturn linked by C—H···O type hydrogen bond results in a molecular chain running along [100] direction.

### S2. Experimental

The chalcone derivative is prepared by two steps.

In a 250 mL round-bottomed flask 4-hydroxyacetophenone (0.05 mol) and 4-methoxybenzaldehyde (0.05 mol) were taken to which 120 mL of absolute alcohol was added and stirred at room temperature for a span of 5 minutes. Then 20 mL of 20% sodium hydroxide solution was added and the mixture was stirred for 2 h. The precipitate generated by adding sufficient amount of dilute hydrochloric acid was filtered, washed with water and dried. The crude product was recrystallized twice from absolute alcohol. % of yield: 90.

The second step involves esterification reaction: in a 250 mL round-bottomed flask the chalcone (0.02 mol) was taken in, to which 120 mL of ethyl methyl ketone was added and stirred at room temperature. After a span of 5 minutes, triethylamine (0.04 mol) was added and the mixture was stirred for 15 minutes. Then benzoylchloride (0.02 mol) was added and the reaction mixture was stirred at room temperature for about 2 h. A white precipitate of triethyl ammonium chloride was formed. It was filtered and the filterate was evaporated to get the crude product. The crude product was recrystallized twice from ethyl methyl ketone solution. % of yield: 95.

### S3. Refinement

H atoms were positioned geometrically and treated as riding on their parent atoms, with C—H distance of 0.93–0.96 Å, with *U*_iso_(H)= 1.5 *U*_eq_(c-methyl) and *U*_iso_(H)= 1.2Ueq(C) for other H atom. The phenyl benzoate group of the molecule was refined using a disorder model, with relative occupancies of approximately 82.3% and 17.7%.

### Crystal data

**Table:**

C_23_H_18_O_4_	*F*(000) = 752
*M**_r_* = 358.37	*D*_x_ = 1.321 Mg m^−^^3^
Monoclinic, *P*2_1_/*c*	Mo *K*α radiation, λ = 0.71073 Å
Hall symbol: -P 2ybc	Cell parameters from 3462 reflections
*a* = 20.146 (5) Å	θ = 2.6–25.6°
*b* = 14.513 (5) Å	µ = 0.09 mm^−^^1^
*c* = 6.187 (5) Å	*T* = 293 K
β = 94.828 (5)°	Block, yellow
*V* = 1802.5 (16) Å^3^	0.35 × 0.30 × 0.25 mm
*Z* = 4	

### Data collection

**Table:**

Bruker APEXII CCD diffractometer	3170 independent reflections
Radiation source: fine-focus sealed tube	2523 reflections with *I* > 2σ(*I*)
Graphite monochromator	*R*_int_ = 0.045
ω and φ scan	θ_max_ = 25.0°, θ_min_ = 1.7°
Absorption correction: multi-scan (*SADABS*; Bruker, 2004)	*h* = −23→23
*T*_min_ = 0.969, *T*_max_ = 0.978	*k* = −17→17
17041 measured reflections	*l* = −7→7

### Refinement

**Table:**

Refinement on *F*^2^	Secondary atom site location: difference Fourier map
Least-squares matrix: full	Hydrogen site location: inferred from neighbouring sites
*R*[*F*^2^ > 2σ(*F*^2^)] = 0.056	H-atom parameters constrained
*wR*(*F*^2^) = 0.195	*w* = 1/[σ^2^(*F*_o_^2^) + (0.0812*P*)^2^ + 1.0118*P*] where *P* = (*F*_o_^2^ + 2*F*_c_^2^)/3
*S* = 1.11	(Δ/σ)_max_ = 0.001
3170 reflections	Δρ_max_ = 0.21 e Å^−^^3^
320 parameters	Δρ_min_ = −0.17 e Å^−^^3^
334 restraints	Extinction correction: *SHELXL*, Fc^*^=kFc[1+0.001xFc^2^λ^3^/sin(2θ)]^-1/4^
Primary atom site location: structure-invariant direct methods	Extinction coefficient: 0.0043 (17)

### Special details

**Table:**

Geometry. All e.s.d.'s (except the e.s.d. in the dihedral angle between two l.s. planes) are estimated using the full covariance matrix. The cell e.s.d.'s are taken into account individually in the estimation of e.s.d.'s in distances, angles and torsion angles; correlations between e.s.d.'s in cell parameters are only used when they are defined by crystal symmetry. An approximate (isotropic) treatment of cell e.s.d.'s is used for estimating e.s.d.'s involving l.s. planes.
Refinement. Refinement of *F*^2^ against ALL reflections. The weighted *R*-factor *wR* and goodness of fit *S* are based on *F*^2^, conventional *R*-factors *R* are based on *F*, with *F* set to zero for negative *F*^2^. The threshold expression of *F*^2^ > σ(*F*^2^) is used only for calculating *R*-factors(gt) *etc*. and is not relevant to the choice of reflections for refinement. *R*-factors based on *F*^2^ are statistically about twice as large as those based on *F*, and *R*- factors based on ALL data will be even larger.

### Fractional atomic coordinates and isotropic or equivalent isotropic displacement parameters (Å^2^)

**Table:**

	*x*	*y*	*z*	*U*_iso_*/*U*_eq_	Occ. (<1)
C1	0.89122 (16)	0.6485 (2)	0.0011 (5)	0.0631 (8)	
H1	0.8506	0.6659	−0.0694	0.076*	
C2	0.94911 (18)	0.6612 (2)	−0.0966 (5)	0.0746 (9)	
H2	0.9474	0.6882	−0.2332	0.090*	
C3	1.00923 (18)	0.6348 (2)	0.0027 (6)	0.0789 (10)	
H3	1.0480	0.6438	−0.0660	0.095*	
C4	1.01200 (17)	0.5948 (3)	0.2058 (6)	0.0773 (9)	
H4	1.0527	0.5760	0.2737	0.093*	
C5	0.95459 (15)	0.5829 (2)	0.3071 (5)	0.0665 (8)	
H5	0.9566	0.5568	0.4448	0.080*	
C6	0.89386 (13)	0.60929 (18)	0.2065 (4)	0.0514 (7)	
C7	0.83392 (14)	0.59524 (18)	0.3243 (5)	0.0540 (7)	
C8	0.71707 (13)	0.62650 (18)	0.3021 (4)	0.0515 (7)	
C9	0.70646 (14)	0.66395 (18)	0.5013 (5)	0.0555 (7)	
H9	0.7417	0.6891	0.5888	0.067*	
C10	0.64306 (14)	0.66356 (18)	0.5684 (5)	0.0555 (7)	
H10	0.6356	0.6871	0.7040	0.067*	
C11	0.59009 (14)	0.62820 (18)	0.4352 (5)	0.0575 (7)	
C12	0.60283 (15)	0.5913 (2)	0.2357 (5)	0.0687 (9)	
H12	0.5679	0.5673	0.1449	0.082*	
C13	0.66607 (15)	0.5899 (2)	0.1713 (5)	0.0625 (8)	
H13	0.6742	0.5641	0.0385	0.075*	
O1	0.83266 (11)	0.55704 (16)	0.4953 (3)	0.0757 (7)	
O2	0.77922 (9)	0.63206 (14)	0.2161 (3)	0.0613 (6)	
C14	0.5205 (2)	0.6314 (5)	0.4965 (9)	0.0649 (15)	0.823 (5)
C15	0.4662 (2)	0.6328 (4)	0.3246 (11)	0.0682 (15)	0.823 (5)
H15	0.4749	0.6488	0.1842	0.082*	0.823 (5)
C16	0.40600 (19)	0.6119 (2)	0.3661 (7)	0.0610 (10)	0.823 (5)
H16	0.3996	0.5946	0.5076	0.073*	0.823 (5)
O4	0.16821 (8)	0.61934 (12)	−0.1635 (3)	0.0731 (16)	0.823 (5)
C20	0.22860 (8)	0.61731 (12)	−0.0591 (3)	0.0506 (9)	0.823 (5)
C21	0.23058 (8)	0.57341 (12)	0.1410 (3)	0.0521 (10)	0.823 (5)
H21	0.1925	0.5454	0.1853	0.063*	0.823 (5)
C22	0.28953 (8)	0.57131 (12)	0.2749 (3)	0.0545 (10)	0.823 (5)
H22	0.2909	0.5419	0.4088	0.065*	0.823 (5)
C17	0.34650 (8)	0.61310 (12)	0.2087 (3)	0.0517 (11)	0.823 (5)
C18	0.34452 (8)	0.65700 (12)	0.0085 (3)	0.0569 (10)	0.823 (5)
H18	0.3826	0.6850	−0.0358	0.068*	0.823 (5)
C19	0.28557 (8)	0.65910 (12)	−0.1254 (3)	0.0567 (11)	0.823 (5)
H19	0.2842	0.6885	−0.2593	0.068*	0.823 (5)
O3	0.5124 (2)	0.6334 (5)	0.6900 (8)	0.0909 (14)	0.823 (5)
C23	0.1593 (5)	0.6736 (9)	−0.3550 (11)	0.073 (2)	0.823 (5)
H23A	0.1704	0.7365	−0.3209	0.109*	0.823 (5)
H23B	0.1137	0.6699	−0.4135	0.109*	0.823 (5)
H23C	0.1878	0.6509	−0.4599	0.109*	0.823 (5)
C14'	0.5326 (7)	0.624 (3)	0.575 (3)	0.063 (6)	0.177 (5)
C15'	0.4574 (6)	0.6085 (10)	0.476 (2)	0.038 (3)	0.177 (5)
H15'	0.4262	0.5844	0.5633	0.046*	0.177 (5)
C16'	0.4385 (10)	0.6305 (14)	0.263 (3)	0.042 (3)	0.177 (5)
H16'	0.4730	0.6518	0.1852	0.050*	0.177 (5)
O4'	0.1697 (4)	0.6196 (6)	−0.1703 (13)	0.060 (6)	0.177 (5)
C20'	0.2427 (4)	0.6185 (6)	−0.0378 (13)	0.062 (4)	0.177 (5)
C21'	0.2604 (4)	0.5803 (6)	0.1649 (13)	0.050 (4)	0.177 (5)
H21'	0.2283	0.5520	0.2417	0.060*	0.177 (5)
C22'	0.3262 (4)	0.5842 (6)	0.2528 (13)	0.045 (4)	0.177 (5)
H22'	0.3380	0.5586	0.3884	0.054*	0.177 (5)
C17'	0.3742 (4)	0.6264 (6)	0.1381 (13)	0.044 (3)	0.177 (5)
C18'	0.3565 (4)	0.6646 (6)	−0.0646 (13)	0.069 (4)	0.177 (5)
H18'	0.3887	0.6929	−0.1413	0.083*	0.177 (5)
C19'	0.2908 (4)	0.6607 (6)	−0.1525 (13)	0.063 (4)	0.177 (5)
H19'	0.2790	0.6863	−0.2881	0.076*	0.177 (5)
O3'	0.5304 (9)	0.628 (2)	0.774 (2)	0.076 (5)	0.177 (5)
C23'	0.161 (3)	0.661 (5)	−0.380 (6)	0.095 (14)	0.177 (5)
H23D	0.1932	0.7093	−0.3896	0.142*	0.177 (5)
H23E	0.1171	0.6853	−0.4042	0.142*	0.177 (5)
H23F	0.1680	0.6149	−0.4884	0.142*	0.177 (5)

### Atomic displacement parameters (Å^2^)

**Table:**

	*U*^11^	*U*^22^	*U*^33^	*U*^12^	*U*^13^	*U*^23^
C1	0.072 (2)	0.0635 (19)	0.0548 (17)	0.0032 (14)	0.0081 (14)	0.0041 (14)
C2	0.089 (3)	0.073 (2)	0.0646 (19)	−0.0015 (17)	0.0248 (18)	0.0038 (16)
C3	0.073 (2)	0.080 (2)	0.088 (2)	−0.0085 (18)	0.0321 (19)	−0.0092 (19)
C4	0.061 (2)	0.088 (2)	0.083 (2)	0.0019 (17)	0.0072 (17)	−0.0069 (19)
C5	0.0627 (19)	0.070 (2)	0.0667 (19)	0.0064 (15)	0.0046 (15)	0.0015 (16)
C6	0.0607 (17)	0.0449 (15)	0.0492 (15)	−0.0006 (12)	0.0076 (12)	−0.0016 (11)
C7	0.0587 (17)	0.0467 (15)	0.0565 (16)	0.0035 (12)	0.0039 (13)	0.0043 (13)
C8	0.0540 (16)	0.0437 (14)	0.0567 (16)	0.0053 (11)	0.0032 (12)	0.0052 (12)
C9	0.0545 (17)	0.0505 (15)	0.0603 (17)	−0.0017 (12)	−0.0031 (13)	−0.0034 (13)
C10	0.0613 (18)	0.0482 (15)	0.0569 (16)	0.0027 (12)	0.0036 (13)	−0.0027 (13)
C11	0.0544 (17)	0.0446 (15)	0.0727 (19)	0.0032 (12)	0.0010 (14)	−0.0049 (13)
C12	0.0598 (19)	0.0595 (18)	0.083 (2)	0.0052 (14)	−0.0134 (16)	−0.0228 (16)
C13	0.067 (2)	0.0611 (18)	0.0575 (17)	0.0132 (14)	−0.0039 (14)	−0.0113 (14)
O1	0.0743 (14)	0.0904 (16)	0.0639 (13)	0.0121 (11)	0.0139 (10)	0.0304 (12)
O2	0.0593 (12)	0.0684 (13)	0.0569 (12)	0.0094 (9)	0.0094 (9)	0.0141 (10)
C14	0.057 (3)	0.059 (3)	0.077 (3)	0.004 (3)	0.001 (3)	−0.009 (3)
C15	0.056 (3)	0.064 (3)	0.087 (4)	0.001 (3)	0.018 (3)	−0.001 (3)
C16	0.065 (2)	0.0484 (19)	0.071 (2)	0.0033 (16)	0.015 (2)	−0.0013 (17)
O4	0.074 (3)	0.082 (3)	0.065 (3)	−0.010 (3)	0.013 (3)	0.005 (3)
C20	0.056 (2)	0.0449 (19)	0.0518 (19)	0.0005 (15)	0.0107 (16)	−0.0033 (16)
C21	0.060 (2)	0.0455 (19)	0.053 (2)	−0.0032 (16)	0.0162 (17)	−0.0025 (15)
C22	0.063 (3)	0.0454 (19)	0.056 (2)	0.0036 (18)	0.013 (2)	0.0043 (16)
C17	0.065 (3)	0.040 (2)	0.051 (2)	0.0039 (19)	0.013 (2)	0.0008 (17)
C18	0.060 (2)	0.048 (2)	0.063 (3)	−0.0060 (16)	0.0084 (18)	0.0041 (18)
C19	0.068 (3)	0.051 (2)	0.053 (2)	−0.001 (2)	0.015 (2)	0.0058 (19)
O3	0.058 (3)	0.125 (3)	0.091 (3)	0.003 (3)	0.013 (2)	−0.003 (3)
C23	0.062 (3)	0.101 (5)	0.056 (4)	−0.007 (3)	0.005 (3)	−0.004 (3)
C14'	0.034 (8)	0.062 (9)	0.086 (12)	0.019 (8)	−0.030 (9)	−0.010 (11)
C15'	0.023 (6)	0.050 (6)	0.044 (6)	−0.002 (5)	0.012 (5)	0.006 (5)
C16'	0.040 (7)	0.041 (6)	0.049 (8)	−0.002 (7)	0.035 (6)	0.006 (6)
O4'	0.054 (10)	0.061 (10)	0.066 (10)	−0.015 (9)	0.018 (9)	0.003 (10)
C20'	0.066 (7)	0.056 (7)	0.064 (7)	−0.012 (6)	−0.001 (6)	−0.012 (7)
C21'	0.047 (8)	0.050 (7)	0.055 (7)	−0.003 (6)	0.020 (6)	−0.003 (6)
C22'	0.053 (8)	0.054 (8)	0.031 (6)	0.007 (7)	0.025 (7)	0.017 (6)
C17'	0.056 (6)	0.039 (6)	0.041 (6)	−0.009 (5)	0.035 (5)	0.007 (5)
C18'	0.095 (9)	0.063 (8)	0.051 (8)	0.007 (7)	0.007 (7)	0.000 (7)
C19'	0.068 (8)	0.054 (8)	0.067 (8)	−0.008 (8)	−0.002 (7)	−0.001 (7)
O3'	0.045 (9)	0.111 (11)	0.070 (11)	0.009 (8)	−0.016 (7)	−0.006 (11)
C23'	0.15 (3)	0.079 (19)	0.051 (17)	0.015 (19)	−0.010 (18)	0.029 (18)

### Geometric parameters (Å, º)

**Table:**

C1—C2	1.371 (4)	C20—C21	1.3900
C1—C6	1.389 (4)	C20—C19	1.3900
C1—H1	0.9300	C21—C22	1.3900
C2—C3	1.366 (5)	C21—H21	0.9300
C2—H2	0.9300	C22—C17	1.3900
C3—C4	1.381 (5)	C22—H22	0.9300
C3—H3	0.9300	C17—C18	1.3900
C4—C5	1.372 (4)	C18—C19	1.3900
C4—H4	0.9300	C18—H18	0.9300
C5—C6	1.379 (4)	C19—H19	0.9300
C5—H5	0.9300	C23—H23A	0.9600
C6—C7	1.476 (4)	C23—H23B	0.9600
C7—O1	1.196 (3)	C23—H23C	0.9600
C7—O2	1.351 (3)	C14'—O3'	1.234 (11)
C8—C13	1.361 (4)	C14'—C15'	1.60 (2)
C8—C9	1.379 (4)	C15'—C16'	1.38 (3)
C8—O2	1.403 (3)	C15'—H15'	0.9300
C9—C10	1.376 (4)	C16'—C17'	1.45 (2)
C9—H9	0.9300	C16'—H16'	0.9300
C10—C11	1.391 (4)	O4'—C23'	1.426 (9)
C10—H10	0.9300	O4'—C20'	1.6236
C11—C12	1.389 (4)	C20'—C21'	1.3900
C11—C14	1.484 (5)	C20'—C19'	1.3900
C11—C14'	1.505 (10)	C21'—C22'	1.3900
C12—C13	1.367 (4)	C21'—H21'	0.9300
C12—H12	0.9300	C22'—C17'	1.3900
C13—H13	0.9300	C22'—H22'	0.9300
C14—O3	1.222 (6)	C17'—C18'	1.3900
C14—C15	1.460 (7)	C18'—C19'	1.3900
C15—C16	1.297 (6)	C18'—H18'	0.9300
C15—H15	0.9300	C19'—H19'	0.9300
C16—C17	1.480 (4)	C23'—H23D	0.9600
C16—H16	0.9300	C23'—H23E	0.9600
O4—C20	1.3287	C23'—H23F	0.9600
O4—C23	1.421 (5)		
			
C2—C1—C6	119.3 (3)	O4—C20—C21	113.5
C2—C1—H1	120.3	O4—C20—C19	126.3
C6—C1—H1	120.3	C21—C20—C19	120.0
C3—C2—C1	121.3 (3)	C22—C21—C20	120.0
C3—C2—H2	119.4	C22—C21—H21	120.0
C1—C2—H2	119.4	C20—C21—H21	120.0
C2—C3—C4	119.6 (3)	C21—C22—C17	120.0
C2—C3—H3	120.2	C21—C22—H22	120.0
C4—C3—H3	120.2	C17—C22—H22	120.0
C5—C4—C3	119.8 (3)	C22—C17—C18	120.0
C5—C4—H4	120.1	C22—C17—C16	116.38 (18)
C3—C4—H4	120.1	C18—C17—C16	123.54 (18)
C4—C5—C6	120.6 (3)	C17—C18—C19	120.0
C4—C5—H5	119.7	C17—C18—H18	120.0
C6—C5—H5	119.7	C19—C18—H18	120.0
C5—C6—C1	119.4 (3)	C18—C19—C20	120.0
C5—C6—C7	118.0 (3)	C18—C19—H19	120.0
C1—C6—C7	122.6 (3)	C20—C19—H19	120.0
O1—C7—O2	123.0 (3)	O3'—C14'—C11	131.7 (18)
O1—C7—C6	125.4 (3)	O3'—C14'—C15'	106.0 (12)
O2—C7—C6	111.6 (2)	C11—C14'—C15'	122.3 (13)
C13—C8—C9	121.3 (3)	C16'—C15'—C14'	120.6 (15)
C13—C8—O2	116.7 (3)	C16'—C15'—H15'	119.7
C9—C8—O2	121.7 (2)	C14'—C15'—H15'	119.7
C10—C9—C8	119.1 (3)	C15'—C16'—C17'	131.3 (16)
C10—C9—H9	120.5	C15'—C16'—H16'	114.3
C8—C9—H9	120.5	C17'—C16'—H16'	114.3
C9—C10—C11	120.5 (3)	C23'—O4'—C20'	120 (2)
C9—C10—H10	119.7	C21'—C20'—C19'	120.0
C11—C10—H10	119.7	C21'—C20'—O4'	127.7
C12—C11—C10	118.6 (3)	C19'—C20'—O4'	112.3
C12—C11—C14	119.3 (3)	C20'—C21'—C22'	120.0
C10—C11—C14	122.1 (3)	C20'—C21'—H21'	120.0
C12—C11—C14'	134.9 (11)	C22'—C21'—H21'	120.0
C10—C11—C14'	105.2 (10)	C17'—C22'—C21'	120.0
C14—C11—C14'	20.7 (7)	C17'—C22'—H22'	120.0
C13—C12—C11	120.8 (3)	C21'—C22'—H22'	120.0
C13—C12—H12	119.6	C18'—C17'—C22'	120.0
C11—C12—H12	119.6	C18'—C17'—C16'	127.7 (8)
C8—C13—C12	119.7 (3)	C22'—C17'—C16'	112.1 (8)
C8—C13—H13	120.1	C17'—C18'—C19'	120.0
C12—C13—H13	120.1	C17'—C18'—H18'	120.0
C7—O2—C8	120.4 (2)	C19'—C18'—H18'	120.0
O3—C14—C15	124.1 (5)	C18'—C19'—C20'	120.0
O3—C14—C11	117.2 (4)	C18'—C19'—H19'	120.0
C15—C14—C11	118.7 (5)	C20'—C19'—H19'	120.0
C16—C15—C14	120.5 (6)	O4'—C23'—H23D	109.5
C16—C15—H15	119.7	O4'—C23'—H23E	109.5
C14—C15—H15	119.7	H23D—C23'—H23E	109.5
C15—C16—C17	125.9 (5)	O4'—C23'—H23F	109.5
C15—C16—H16	117.0	H23D—C23'—H23F	109.5
C17—C16—H16	117.0	H23E—C23'—H23F	109.5
C20—O4—C23	117.6 (4)		
			
C6—C1—C2—C3	0.9 (5)	C23—O4—C20—C21	171.5 (7)
C1—C2—C3—C4	−0.2 (5)	C23—O4—C20—C19	−3.9 (7)
C2—C3—C4—C5	−0.8 (5)	O4—C20—C21—C22	−175.8
C3—C4—C5—C6	1.0 (5)	C19—C20—C21—C22	0.0
C4—C5—C6—C1	−0.2 (5)	C20—C21—C22—C17	0.0
C4—C5—C6—C7	−179.6 (3)	C21—C22—C17—C18	0.0
C2—C1—C6—C5	−0.8 (4)	C21—C22—C17—C16	176.82 (17)
C2—C1—C6—C7	178.6 (3)	C15—C16—C17—C22	167.8 (4)
C5—C6—C7—O1	−5.3 (4)	C15—C16—C17—C18	−15.6 (5)
C1—C6—C7—O1	175.3 (3)	C22—C17—C18—C19	0.0
C5—C6—C7—O2	174.1 (2)	C16—C17—C18—C19	−176.58 (18)
C1—C6—C7—O2	−5.3 (4)	C17—C18—C19—C20	0.0
C13—C8—C9—C10	0.4 (4)	O4—C20—C19—C18	175.2
O2—C8—C9—C10	174.3 (2)	C21—C20—C19—C18	0.0
C8—C9—C10—C11	−1.7 (4)	C12—C11—C14'—O3'	150 (3)
C9—C10—C11—C12	1.6 (4)	C10—C11—C14'—O3'	−16 (4)
C9—C10—C11—C14	−176.5 (4)	C14—C11—C14'—O3'	−163 (8)
C9—C10—C11—C14'	170.5 (15)	C12—C11—C14'—C15'	−27 (4)
C10—C11—C12—C13	−0.1 (5)	C10—C11—C14'—C15'	166 (2)
C14—C11—C12—C13	178.0 (4)	C14—C11—C14'—C15'	19 (2)
C14'—C11—C12—C13	−164.9 (18)	O3'—C14'—C15'—C16'	159 (2)
C9—C8—C13—C12	1.0 (4)	C11—C14'—C15'—C16'	−23 (4)
O2—C8—C13—C12	−173.1 (3)	C14'—C15'—C16'—C17'	−177 (2)
C11—C12—C13—C8	−1.2 (5)	C23'—O4'—C20'—C21'	−179 (3)
O1—C7—O2—C8	−0.2 (4)	C23'—O4'—C20'—C19'	0 (3)
C6—C7—O2—C8	−179.6 (2)	C19'—C20'—C21'—C22'	0.0
C13—C8—O2—C7	−126.4 (3)	O4'—C20'—C21'—C22'	178.8
C9—C8—O2—C7	59.5 (3)	C20'—C21'—C22'—C17'	0.0
C12—C11—C14—O3	155.5 (5)	C21'—C22'—C17'—C18'	0.0
C10—C11—C14—O3	−26.5 (8)	C21'—C22'—C17'—C16'	175.2 (10)
C14'—C11—C14—O3	12 (4)	C15'—C16'—C17'—C18'	167.7 (15)
C12—C11—C14—C15	−25.0 (7)	C15'—C16'—C17'—C22'	−7 (2)
C10—C11—C14—C15	153.0 (5)	C22'—C17'—C18'—C19'	0.0
C14'—C11—C14—C15	−169 (5)	C16'—C17'—C18'—C19'	−174.4 (11)
O3—C14—C15—C16	−18.4 (10)	C17'—C18'—C19'—C20'	0.0
C11—C14—C15—C16	162.1 (5)	C21'—C20'—C19'—C18'	0.0
C14—C15—C16—C17	178.2 (4)	O4'—C20'—C19'—C18'	−178.9

### Hydrogen-bond geometry (Å, º)

**Table:**

*D*—H···*A*	*D*—H	H···*A*	*D*···*A*	*D*—H···*A*
C21—H21···O1^i^	0.93	2.56	3.280 (3)	135
C21′—H21′···O1^i^	0.93	2.65	3.546 (7)	163
C23′—H23*F*···O1^ii^	0.96	2.50	3.24 (7)	135

Symmetry codes: (i) −*x*+1, −*y*+1, −*z*+1; (ii) −*x*+1, −*y*+1, −*z*.
