# Supplementary figures and images for: Crystal structure of 4-[(2E)-3-(4-meth­oxy­phen­yl)prop-2-eno­yl]phenyl benzoate
Source: Acta Crystallogr Sect E Struct Rep Online. 2014 Aug 16;70(Pt 9):o1007. doi: 10.1107/S1600536814018303 (PMC4186117; doi:10.1107/S1600536814018303)

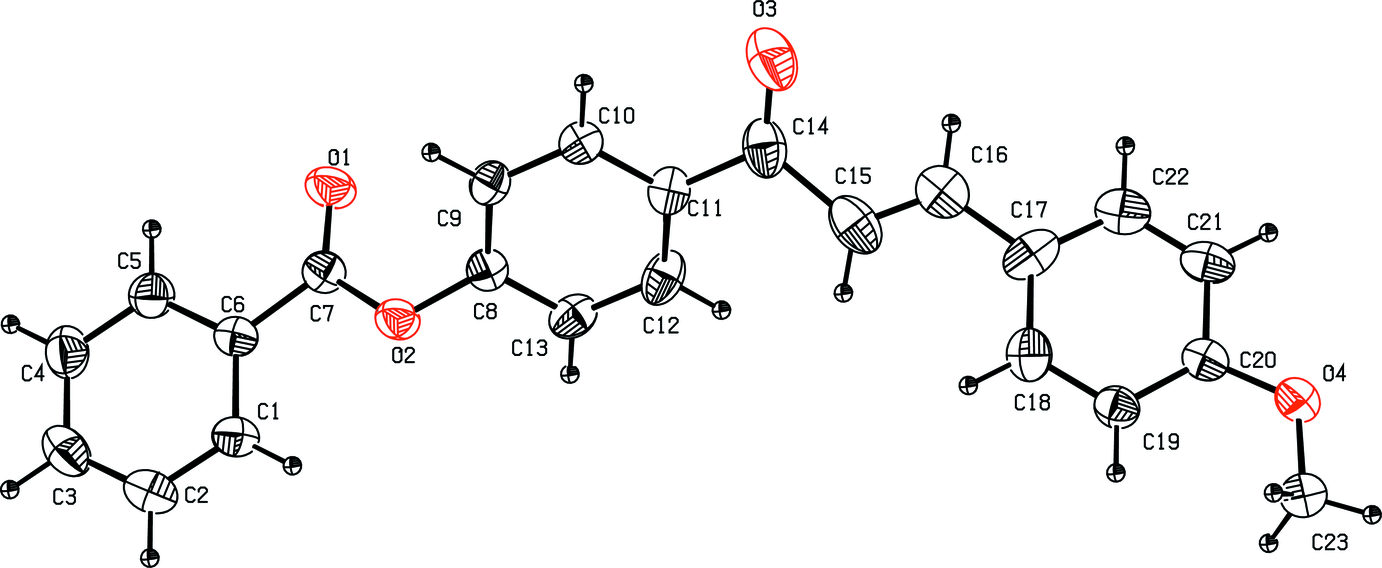

Supplement: Supplementary file 4 [file e-70-o1007-fig1.tif]

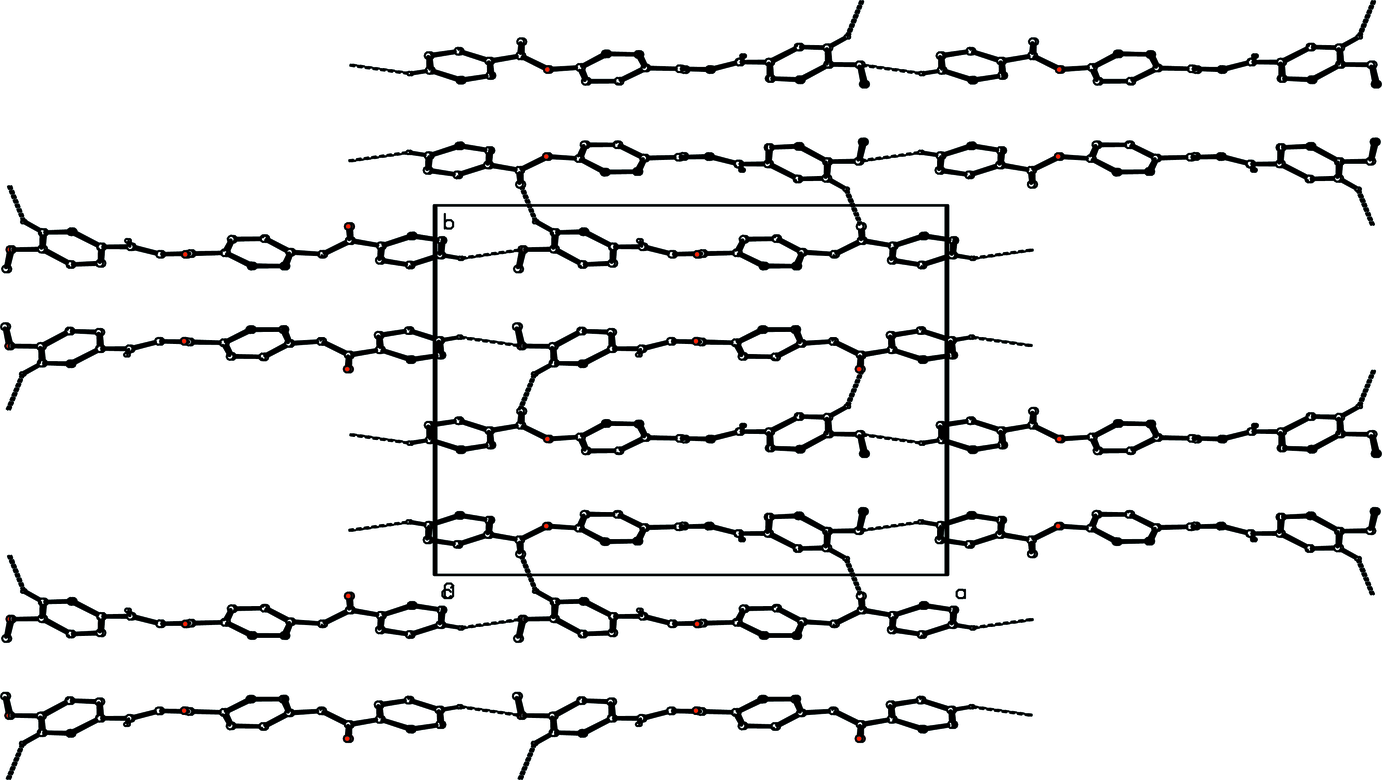

Supplement: Supplementary file 5 [file e-70-o1007-fig2.tif]
